# Elevation of the head of bed reduces splanchnic blood flow in patients with intra-abdominal hypertension

**DOI:** 10.1186/s12871-023-02046-8

**Published:** 2023-04-22

**Authors:** Yuankai Zhou, Huaiwu He, Na Cui, Xiaoting Wang, Yun Long, Dawei Liu

**Affiliations:** Department of Critical Care Medicine, State Key Laboratory of Complex Severe and Rare Diseases, Peking Union Medical College, Peking Union Medical College Hospital, Chinese Academy of Medical Sciences, Beijing, 100730 China

**Keywords:** Head-up positioning, Intra-abdominal hypertension, Celiac artery, Superior mesenteric artery, Splanchnic blood flow, Doppler ultrasound

## Abstract

**Background:**

Elevation of the head of bed (HOB) increases intra-abdominal pressure (IAP), but the effect of body position on abdominal splanchnic perfusion is not clear. The current study aimed to evaluate the effect of body position on the superior mesenteric artery (SMA) and the celiac artery (CA) blood flow by Doppler ultrasound in mechanically ventilated patients with intra-abdominal hypertension (IAH).

**Methods:**

This prospective cohort study included 53 mechanically ventilated patients with IAH. IAP, hemodynamic variables, and Doppler parameters of the SMA and CA were measured in the supine position. The measurements were repeated after the HOB angle was raised to 15° for 5 min and similarly at HOB angles of 30° and 45°. Finally, the patient was returned to the supine and these variables were re-measured.

**Results:**

The median (interquartile range, IQR) superior mesenteric artery blood flow (SMABF) decreased from 269 (244–322) to 204 (183–234) mL/min and the median (IQR) celiac artery blood flow (CABF) from 424 (368–483) to 376 (332–472) mL/min (both p<0.0001) while median (IQR) IAP increased from 14(13–16) to 16(14–18) mmHg (p<0.0001) when the HOB angle was changed from 0° to 15°. However, SMABF and CABF were maintained at similar levels from 15° to 30°, despite median (IQR) IAP increased to 17(15–18) mmHg (p = 0.0002). Elevation from 30° to 45° further reduced median (IQR) SMABF from 200(169–244) to 164(139–212) mL/min and CABF from 389(310–438) to 291(241–383) mL/min (both p<0.0001), Meanwhile, median (IQR) IAP increased to 19(18–21) mmHg (p<0.0001).

**Conclusions:**

In mechanically ventilated patients with IAH, progressive elevation of the HOB from a supine to semi-recumbent position was associated with a gradual reduction in splanchnic blood flow. However, the results indicate that splanchnic blood flow is not further reduced when the HOB is elevated from 15° to 30°.This study confirms the influence of head-up angle on blood flow of the splanchnic organs and may contribute to the selection of the optimal position in patients with abdominal hypertension.

**Supplementary Information:**

The online version contains supplementary material available at 10.1186/s12871-023-02046-8.

## Background

Intra-abdominal hypertension (IAH) is a frequent cause of morbidity and mortality among critically ill patients [1–3]. IAH leads to the decrease of intestinal perfusion, cause intestinal mucosal damage and sepsis of intestinal origin. The cut-off value used to define IAH in patients is pressure above 12 mmHg. A lot of risk factors for IAH has been described, and they can be divided into three categories: increased intra-abdominal volume, decreased abdominal compliance, and a combination of both [1]. There have been a few studies on the relationship between the head of bed (HOB) position and intra-abdominal pressure (IAP) [4–6]. These results indicate that raising the HOB angle significantly increases IAP and decreases abdominal perfusion pressure (APP). All guidelines for the treatment of IAH mention the impact of body position and recommend the supine position to improve abdominal wall compliance [1, 2]. However, for mechanically ventilated patients, the position recommended by the guidelines is semi-recumbent (45°) to reduce the incidence of ventilator-associated pneumonia (VAP) [7, 8].

The available reports on the influence of body position have focused on monitoring distinct pressure indicators, such as IAP and APP. According to Poiselle’s law, flow = pressure/resistance. Therefore, a decrease in APP does not necessarily lead to the completely consistent decrease in blood flow of abdominal organs [9]. Although non-occlusive mesenteric ischemia (NOMI) caused by decreased splanchnic arterial flow is a life-threatening condition leading to intestinal ischemia and multi‑organ failure[10, 11], there is still a lack of point-of-care monitoring of splanchnic blood flow.

This study evaluated whether elevation of the HOB reduces blood flow in the celiac artery(CA) and superior mesenteric artery(SMA) using Doppler ultrasound.

## Materials and methods

### Patient enrollment

This prospective study was performed based on the Declaration of Helsinki. All experiments were performed in accordance with relevant guidelines and regulations. All the study protocols were approved by the Institutional Research and Ethics Committee of the Peking Union Medical College Hospital for human subjects (Ethics certificate number: ZS-3088). Informed consent was obtained from the next of kin or patients before enrollment in the study.

The prospective cohort trial was performed in the Critical Care Department of Peking Union Medical College Hospital in China. Mechanically ventilated patients who were in the intensive care unit (ICU) were enrolled upon being diagnosed with IAH.

The inclusion criteria were as follows: (1) tracheal intubation with mechanical ventilation and sedation; (2) bladder pressure ≥ 12 mmHg; (3) age 18–80 years; (4) sinus rhythm.

The exclusion criteria were as follows: (1) coronary heart disease, severe mesenteric stenosis, or celiac artery stenosis; (2) patients in a fixed position (such as recent spinal surgery or intracranial hypertension); (3) patients for whom IAP measurements were contraindicated (e.g., those with recent bladder surgery, injury, or pregnancy); (4) patients who had underwent abdominal surgeries involving the intestine, the descending thoracic or abdominal aortic procedures; (5) poor quality of abdominal ultrasound images; (6) pleural fluid or ascites.

### Study design

This study was initiated to determine the specific head of bed angle elevation that had the least impact on the blood flow of the internal organs compared to that measured in the supine position. In the pre-experimental stage, the blood flow of the CA (CABF) and superior mesenteric artery (SMABF) was measured at HOB angles of 0° and 15° in 18 patients with the same demographics, and the blood flow difference between two HOB angles was calculated. The study was designed with 80% power to detect a minimum blood flow difference between two angles with a two-tailed alpha of 0.05. A power calculation indicated that 21 patients were required to detect a difference in CABF and SMABF, and 53 patients were included in the study.

### Study protocol

Epidemiological data pertaining to the patients were studied upon admission. The patients were sedated to a Richmond Agitation Sedation Score (RASS) of −4 during the time of acquiring the measurements as per the World Society of the Abdominal Compartment Syndrome consensus recommendations [2]. Only sedative drugs and opioid analgesics were used, and no muscular relaxants were administered.

Pulse index continuous cardiac output (PiCCO) (PV2015L20A;Pulsion Medical Systems, Feldkirchen, Germany) was used to monitor cardiac output. The peripheral perfusion index (PPI) is an indicator of peripheral perfusion derived from the photoelectric plethysmographic signal of a pulse oximeter. It is defined as the ratio between the pulsatile and non-pulsatile portions of the plethysmographic waveform. The PPI was assessed using a Philips Medical Systems Viridia/56S monitor.

**Fig. 1:**
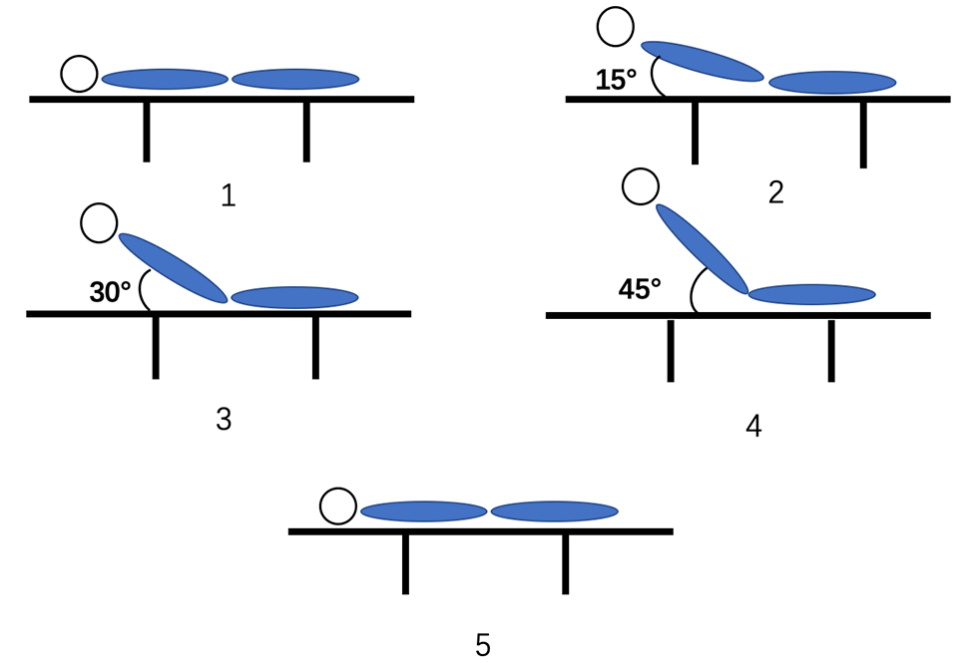
Study protocol

IAP, systemic hemodynamic variables, and the Doppler parameters of the SMA and CA were measured at the HOB of 0°, 15°, 30°, 45°. Parameters were measured 5 min after each angle elevation, in order to stabilize the hemodynamic parameters. The time interval between each head up movement is 5 min plus the measurement time, which is about 15 min.Thereafter, the HOB angle was restored to 0° and measurements were conducted (Fig. 1).

Blood pressure was supported by infusion of noradrenaline and aimed to maintain the blood pressure recorded at baseline in the supine position.

Standard blood samples of 2 mL were collected from the central venous catheter and the line of radial artery concurrently. All blood samples were examined by a Radiometer ABL800 FLEX analyzer (Radiometer Medical ApS, Copenhagen, Denmark). The central venous blood sample was analyzed to measure central venous oxygen saturation(ScvO_2_). Central venous-to-arterial carbon dioxide tension difference(Pv-aCO_2_) was calculated as the difference between the central venous CO_2_ tension and arterial CO_2_ tension.

### IAP monitoring

IAP was evaluated indirectly by measuring bladder pressure. It was measured using the intravesicular technique (Beijing Winsunny Harmony Science & Technology Co., Ltd.) with a standard instillation volume of 20 mL of sterile saline according to the recommendations of the World Society of the Abdominal Compartment Syndrome consensus [2]. After the patient was enrolled, the point corresponding to the midaxillary line at the level of the iliac crest was marked on the patient’s skin as the zero reference point for all subsequent IAP measurements. IAP was expressed in mmHg and measured at the end of expiration, thereby ensuring that abdominal muscle contractions were absent. A waiting period of 30s was included to allow relaxation of the bladder detrusor muscle. The instilled saline was completely drained from the bladder before the next IAP measurement. The IAP was measured twice at each body position, the interval between the two measurements was 3 min, and the mean of the two results was used for calculations.

### Doppler ultrasound monitoring

Doppler ultrasound was performed with an ultrasound system consisting of a 2–5 MHz C60xp Probe (X-Porte Ultrasound System, FUJIFILM SONOSITE, INC., USA). Blood flow parameters of each vessel were measured by two ICU physicians, and the average value was recorded. Each of the two ICU physicians had more than four years of experience in point of care ultrasound and obtained their certification from the Chinese Critical Ultrasound Study Group.

The CA flow was obtained 1 cm proximal to the abdominal aorta before its division into the hepatic and splenic arteries. SMA flow was measured 1 cm proximal to the abdominal aorta. The angle of insonation was < 60°.

The Doppler parameters were measured in the longitudinal section of the vessel, and the diameter(D) of the vessel was measured in cross-section at the same location.

To reduce the error, the average of three measurements was taken in the zoomed state and the cross-sectional was calculated.

The time–velocity waveform readings were used to measure time-averaged mean velocity(TAMV), and blood flow (BF, mL/min), according to the calculation software carried by the ultrasound. BF = π(D/2)²×TAMV×60. The calculation formula of APP: APP = MAP-IAP.

### Statistical analysis

A descriptive analysis was performed. Continuous variables were expressed as the means ± standard deviation or median and interquartile range. Normal distribution was evaluated by Kolmogorov-Smirnov test. Repeated measures one-way ANOVA was used to evaluate variables with normal distribution, and if the test was significant, paired t-tests were used to evaluate changes between different angles of the HOB with Bonferroni correction for multiple comparisons. Variables with non-parametric distributions, the Wilcoxon matched-pairs signed rank test and Friedman test was used. The 2-tailed significance level was set at p < 0.05. Statistical analysis was performed using SPSS Statistics 25.0 (IBM Analytics, USA).

## Results

A total of 66 patients with IAH between December 2020 and February 2022 met the inclusion criteria. Among these patients, six were excluded as they had underwent abdominal intestinal surgery, three due to cardiac arrhythmias emerging during the study, and four because of poor quality of abdominal ultrasound images. Thus, 53 patients were finally included in the study.

The demographics, basic hemodynamic parameters, and causes of abdominal hypertension are presented in Table 1. The hemodynamic parameters, IAP, and ultrasound measurements for each body position are summarized in Table 2.

**Table 1:** Characteristics of the patients

Variable	n = 53
Sex, n, female/male	33/20
Age, years	56 ± 13
BMI (kg/m^2^)	25.6 ± 2.3
CVP (mmHg)	10(9–11)
PH	7.40(7.38–7.42)
PaO_2_/FiO_2_ (mmHg)	215(193–292)
ScvO_2_ (%)	69.9 ± 5.9
Pv-aCO_2_ (mmHg)	4 ± 2
Lactate (mmol/L)	1.7(1.2–2.6)
SOFA	10(9–11)
Vasopressor support, n (%)	46 (86.8)
Cause of Intra-abdominal hypertension	
Complications after surgery^a^, n (%)	35 (66.0)
Sepsis^b^, n (%)	14 (22.7)
colon obstruction, n (%)	3 (5.7)
pancreatitis, n (%)	1(1.9)

Continuous data are expressed as mean ± standard deviation or median and interquartile range

Abbreviations: BMI, body mass index; CVP, central venous pressure; PH, hydrogen ion concentration; PaO_2_, partial pressure of oxygen in arterial blood; FiO_2_, fraction of inspired oxygen; ScvO_2_, superior vena cava oxygen saturation; Pv-aCO_2_, arterial and venous carbon dioxide partial pressure difference; SOFA, sequential organ failure assessment

^a^ Surgery (35): heart valve replacement surgery (19), pericardial stripping surgery (5), esophageal cancer surgery (2), severe multiple trauma(3), neurosurgery(4), descending colon surgery(2); ^b^ Sepsis (14): severe pneumonia(11), biliary infection(3)

**Table 2:** Parameters of different angle of head of bed(HOB)

Variables	0°	15°	30°	45°	*p* ^*a*^
CI(L/min/m^2^)	2.3(2.1–2.5)	2.1(1.9–2.4)*	2.0(1.8-2.0)*†	1.8(1.7–2.1)*†‡	< 0.0001
Heart Rate(bpm)	88(79–95)	88(79–97)	90(80–96)*†	92(81–98)*†	< 0.0001
MAP(mmHg)	75 ± 6	75 ± 6	76 ± 6	76 ± 6	0.06
NE(µg/kg/min)	0.086 ± 0.067	0.088 ± 0.068	0.090 ± 0.069	0.090 ± 0.067	0.55
IAP(mmHg)	14(13–16)	16(14–18)*	17(15–18)*†	19(18–21)*†‡	< 0.0001
APP(mmHg)	61 ± 6	59 ± 6*	59 ± 6*	57 ± 6*†‡	< 0.0001
SMA-TAMV (cm/s)	20.0(17.4–24.0)	15.9 (12.9–19.4)*	15.2(13.2–20.1)*	14.2(11.3–17.7)*†‡	< 0.0001
SMA-D(cm)	0.54(0.52–0.57)	0.53(0.51–0.56)	0.53(0.50–0.55)*†	0.51(0.49–0.53)*†‡	< 0.0001
SMABF(ml/min)	269(244–322)	204(183–234)*	200(169–244)*	164(139–212)*†‡	< 0.0001
SMABF/CO(%)	6.9(6.4–7.6)	5.7(5.0-6.4)*	5.9(5.1–6.8)*	5.1(4.5–6.4)*†‡	< 0.0001
CABF(ml/min)	424(368–483)	376(332–472)*	389(310–438)*	291(241–383)*†‡	< 0.0001
CABF/CO(%)	10.8 ± 2.0	10.6 ± 2.1	10.8 ± 2.6	9.5 ± 2.6*†‡	< 0.0001
PPI	1.60(0.79–2.76)	1.30(0.74–2.50)*	1.20(0.63–2.10)*†	1.10(0.40–1.60)*†‡	< 0.0001

The data are presented as mean ± standard deviation or median and interquartile range

Abbreviations: CI, cardiac index; MAP, mean arterial pressure; NE, norepinephrine; IAP, intra-abdominal pressure; APP, abdominal perfusion pressure; SMA-TAMV, time-averaged mean velocity of the superior mesenteric artery; SMA-D, diameter of the superior mesenteric artery; SMABF, superior mesenteric artery blood flow; CABF, celiac artery blood flow; PPI, peripheral perfusion index

* *p* < 0.05 compared with HOB elevation 0°

† *p* < 0.05 compared with HOB elevation 15°

‡ *p* < 0.05 compared with HOB elevation 30°

*p*^a^: Friedman test or repeated measures one-way ANOVA; multiple comparisons with Bonferroni correction between 0°, 15°, 30° and 45°

The results showed that cardiac index (CI) significantly decreased with each elevation of HOB while MAP and NE infusion rate were maintained at all angles (Table 2).

The IAP increased progressively when the HOB was elevated from 0° to 45°. HOB elevation was associated with significant decreases in PPI.

SMABF and CABF both decreased when the HOB was raised to 15° from the supine position(both *p*<0.0001). However, from 15° to 30°, SMABF (*p* = 0.2032) and CABF ( *p* = 0.0533) remained reduced at the same level. The elevation of the HOB angle from 30° to 45° was associated with a further decrease in SMABF and CABF (both *p*<0.0001) (see Table 2 and Fig. S1). Similar changes were observed for APP.

The diameter of the SMA(SMA-D) and the time-averaged mean velocity of the superior mesenteric artery (SMA-TAMV) decreased with an elevation in the HOB angle (see Table 2).

As mentioned above, the CI decreased when the HOB was elevated. However, from supine to 15° and from 30° to 45°, the proportion of SMABF of CO (SMABF/CO) decreased significantly. At the same time, the ratio of CABF of CO (CABF/CO) was generally stable until 45° (see Table 2).

After the HOB elevation was completed, the patient was restored to the supine position, and the parameters were re-measured as reset values (shown in step 5 in Fig. 1). There was no significant statistical difference between the reset and baseline values in the supine position (Table S1).

## Discussion

In this study, we investigated the association between the head-up angle and SMABF and CABF with Doppler ultrasound technology in mechanically ventilated patients with IAH. Our main findings were as follows: (1) The elevation of the HOB caused an increase in IAP and a decrease in the blood flow of the SMA and CA, particularly at an elevation of 45°. (2) There was no significant change in SMA and CA blood flow between 15° and 30°.

Although the semi-recumbent position is recommended to reduce the incidence of VAP [7], some studies have also found that a slightly lower raising angle could also have the same effect [8]. Therefore, it is important to find a HOB angle that has the least impact on the blood flow of the splanchnic artery.

According to the present study, the degree of HOB elevation is related to the increase in intra-abdominal pressure. Cheatham et al. [4] found that both IAP at 15° and 30° were significantly increased compared with IAP at supine in critically ill medical and surgical patients at risk for intra-abdominal hypertension. Vasquez et al.[12] found the IAP increased progressively when the HOB was elevated (0°, 15°, 30°, and 45°) in 45 trauma patients. Yi’s research had similar conclusion [6].

The present study confirms that elevation of HOB in patients with IAH reduces splanchnic blood flow. Interestingly, our study indicated that SMA and CA blood flow was unaffected when the HOB was elevated from 15° to 30°.

This phenomenon may be due to the fact that there was no statistical difference in APP between HOB 15° and 30°, suggesting no changes in perfusion pressure of the abdominal organs. This study supports that the IAH patient should be placed in the supine position and that if elevation of HOB is required it should not exceed 30°.

As mentioned above, most of the studies on head-up positioning of critically ill patients monitored pressure indicators such as IAP or APP [4–6, 12]. However, this method ignores the autonomous regulation of splanchnic organs and does not directly monitor changes in organ blood flow. Evaluation of splanchnic blood flow using Doppler ultrasound might prove helpful to guide treatment in this population but more research is needed.

Doppler ultrasound can monitor the blood flow of the splanchnic arteries at the bedside in real-time and non-invasively. As an important digestive and immune organ, the intestine plays an important role in the pathophysiological process of sepsis or multiple organ dysfunction syndrome. Further studies on intestinal blood flow and function are required.

### Limitations

(1) The standard deviation of the data was large owing to the large individual differences in the SMABF. This may have affected the statistical results. (2) Doppler measurements could have been affected by subjective factors of the person performing the measurement although one previous study has reported that the blood flow of SMA measured by Doppler ultrasound has good repeatability [13]. (3) The effect of different HOB angles on the prognosis of IAH patients has not been studied. Therefore, further follow-up studies are required. (4) IAP was not measured directly, rather it was estimated by measuring bladder pressure. (5) The population of this study was mechanically ventilated and sedated IAH patients. The results may not be applicable to other patient populations or patients that are not mechanically ventilated.

## Conclusion

In mechanically ventilated patients with IAH, progressive elevation of the HOB from a supine to semi-recumbent position was associated with a gradual reduction in splanchnic blood flow. However, the results indicate that splanchnic blood flow is not further reduced when the HOB is elevated from 15° to 30°.This study confirms the influence of head-up angle on blood flow of the splanchnic organs and may contribute to the selection of the optimal position in patients with abdominal hypertension.

## Electronic supplementary material

Below is the link to the electronic supplementary material.

Supplementary Material 1

Supplementary Material 2

Supplementary Material 3

## Data Availability

The data generated and analyzed during this study are not publicly available due to the protection for the patients’ privacy but are available from the corresponding author on reasonable request.

## Abbreviations

APP: abdominal perfusion pressure
CA: celiac artery
CABF: celiac artery blood flow
CI: cardiac index
CO: cardiac output
CPB: cardiopulmonary bypass
CVP: central venous pressure
EDV: end-diastolic velocity
HOB: head of bed
HR: heart rate
IAH: intra-abdominal hypertension
IAP: intra-abdominal pressure
NE: norepinephrine
MAP: mean arterial pressure
PPI: peripheral perfusion index
SMA: superior mesenteric artery
SMABF: superior mesenteric artery blood flow
ScvO_2_: central venous oxygen saturation
SOFA: sequential organ failure assessment
TAMV: time-averaged mean velocity
