# Supplementary material for: Elevation of the head of bed reduces splanchnic blood flow in patients with intra-abdominal hypertension
Source: BMC Anesthesiol. 2023 Apr 22;23:133. doi: 10.1186/s12871-023-02046-8 (PMC10122394; doi:10.1186/s12871-023-02046-8)
Supplement: Supplementary file 1 — Supplementary Material 1 [file 12871_2023_2046_MOESM1_ESM.docx]

Table S1 Comparison of parameters in the supine position between the baseline state and the recovery state

| Variables | 0° | 0°-reset | *p* |
| --- | --- | --- | --- |
| CI(L/min) | 2.3(2.1-2.5) | 2.3(2.1-2.6) | 0.41 |
| Heart Rate(bpm) | 88(79-95) | 88(80-94) | 0.95 |
| MAP(mmHg) | 75±6 | 75±6 | 0.77 |
| PEEP(cmH_2_O) | 10(10-12) | 10(10-12) | 1.00 |
| Pplat (cmH_2_O) | 25±3 | 25±2 | 0.81 |
| NE(μg/kg/min) | 0.086±0.067 | 0.085±0.068 | 0.53 |
| IAP(mmHg) | 14(13-16) | 14(13-16) | 0.06 |
| APP(mmHg) | 61±6 | 61±6 | 0.73 |
| SMA-TAMV (cm/s) | 20.0(17.4-24.0) | 20.9(16.7-24.6) | 0.99 |
| SMA-D(cm) | 0.54(0.52-0.57) | 0.54(0.52-0.58) | 0.42 |
| SMABF(ml/min) | 269(244-322) | 274(239-328) | 0.60 |
| SMABF/CO(%) | 6.9(6.4-7.6) | 7.1(6.4-7.8) | 0.83 |
| CABF(ml/min) | 424(368-483) | 434(373-493) | 0.64 |
| CABF/CO(%) | 10.8±2.0 | 10.8±2.0 | 0.87 |
| PPI | 1.60(0.79-2.76) | 1.60(0.81-2.65) | 0.44 |

The data are presented as mean± standard deviation or median and interquartile range.

CI, cardiac index; CO, cardiac output; MAP, mean arterial pressure; PEEP, positive end-expiratory pressure; Pplat, plateau pressure; NE, norepinephrine; IAP, intra-abdominal pressure; APP, abdominal perfusion pressure; SMA-TAMV, time-averaged mean velocity of the superior mesenteric artery; SMA-D, diameter of the superior mesenteric artery; SMABF, superior mesenteric artery blood flow; CABF, celiac artery blood flow; PPI, peripheral perfusion index.

*p*: paired t-test or Wilcoxon test.
