# Supplementary figures and images for: Elevation of the head of bed reduces splanchnic blood flow in patients with intra-abdominal hypertension
Source: BMC Anesthesiol. 2023 Apr 22;23:133. doi: 10.1186/s12871-023-02046-8 (PMC10122394; doi:10.1186/s12871-023-02046-8)

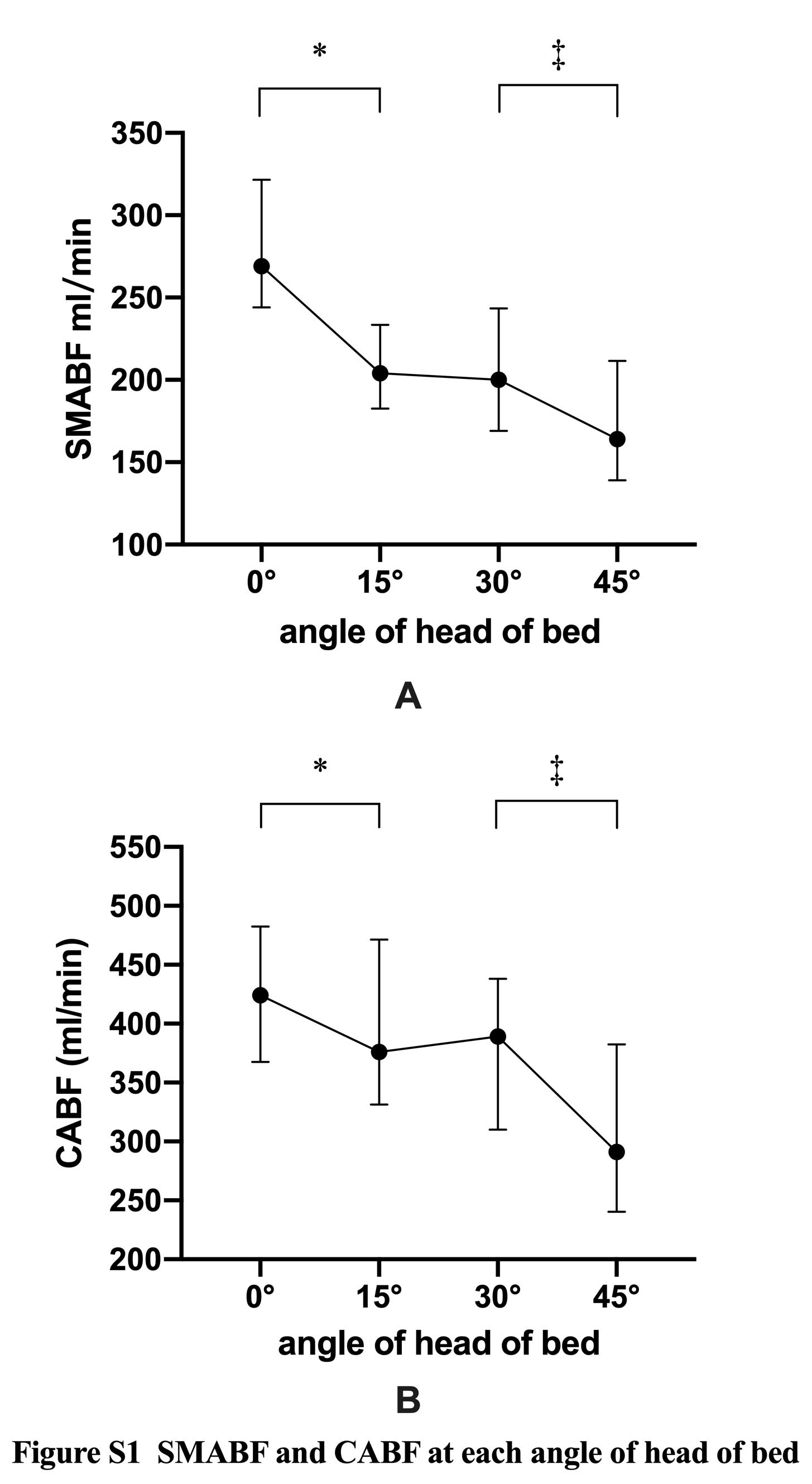

Supplement: Supplementary file 2 — Supplementary Material 2 [file 12871_2023_2046_MOESM2_ESM.tiff]
