# Supplementary material for: Elevation of the head of bed reduces splanchnic blood flow in patients with intra-abdominal hypertension
Source: BMC Anesthesiol. 2023 Apr 22;23:133. doi: 10.1186/s12871-023-02046-8 (PMC10122394; doi:10.1186/s12871-023-02046-8)
Supplement: Supplementary file 3 — Supplementary Material 3 [file 12871_2023_2046_MOESM3_ESM.docx]

[Figure Legends]

Fig.S1 SMABF and CABF at each angle of head of bed

SMABF: superior mesenteric artery blood flow

CABF: celiac artery blood flow

* *p*< 0.05 compared with HOB elevation 0°

‡ *p*< 0.05 compared with HOB elevation 30°
